# Gastrin: A Distinct Fate of Neurogenin3 Positive Progenitor Cells in the Embryonic Pancreas

**DOI:** 10.1371/journal.pone.0070397

**Published:** 2013-08-05

**Authors:** Yaron Suissa, Judith Magenheim, Miri Stolovich-Rain, Ayat Hija, Patrick Collombat, Ahmed Mansouri, Lori Sussel, Beatriz Sosa-Pineda, Kyle McCracken, James M. Wells, R. Scott Heller, Yuval Dor, Benjamin Glaser

**Affiliations:** 1 Endocrinology and Metabolism Service, Department of Internal Medicine, Hadassah-Hebrew University Medical Center, Jerusalem, Israel; 2 Department of Developmental Biology and Cancer Research, The Institute for Medical Research Israel-Canada, The Hebrew University-Hadassah Medical School, Jerusalem, Israel; 3 Department of Diabetes Genetics, Inserm, Nice, France; 4 University of Nice Sophia Antipolis, UFR Sciences, Nice, France; 5 Department of Molecular Cell Biology, Max-Planck Institute for Biophysical Chemistry, Gottingen, Germany; 6 Department of Genetics and Development, Columbia University, New York, New York, United States of America; 7 Department of Genetics, St. Jude Children's Research Hospital, Memphis, Tennessee, United States of America; 8 Division of Developmental Biology, Cincinnati Children's Hospital Medical Center, Cincinnati, Ohio, United States of America; 9 Histology and Delivery Department, Novo Nordisk, Måløv, Denmark; University of British Columbia, Canada

## Abstract

Neurogenin3^+^ (Ngn3^+^) progenitor cells in the developing pancreas give rise to five endocrine cell types secreting insulin, glucagon, somatostatin, pancreatic polypeptide and ghrelin. Gastrin is a hormone produced primarily by G-cells in the stomach, where it functions to stimulate acid secretion by gastric parietal cells. Gastrin is expressed in the embryonic pancreas and is common in islet cell tumors, but the lineage and regulators of pancreatic gastrin^+^ cells are not known. We report that gastrin is abundantly expressed in the embryonic pancreas and disappears soon after birth. Some gastrin^+^ cells in the developing pancreas co-express glucagon, ghrelin or pancreatic polypeptide, but many gastrin^+^ cells do not express any other islet hormone. Pancreatic gastrin^+^ cells express the transcription factors Nkx6.1, Nkx2.2 and low levels of Pdx1, and derive from Ngn3^+^ endocrine progenitor cells as shown by genetic lineage tracing. Using mice deficient for key transcription factors we show that gastrin expression depends on Ngn3, Nkx2.2, NeuroD1 and Arx, but not Pax4 or Pax6. Finally, gastrin expression is induced upon differentiation of human embryonic stem cells to pancreatic endocrine cells expressing insulin. Thus, gastrin^+^ cells are a distinct endocrine cell type in the pancreas and an alternative fate of Ngn3+ cells.

## Introduction

The islets of Langerhans are composed of 4 main endocrine cell types: beta cells secreting insulin, alpha cells secreting glucagon, delta cells secreting somatostatin, and PP cells secreting pancreatic polypeptide. These cells all derive from endocrine progenitor cells in the embryonic pancreas, marked by expression of the transcription factor neurogenin3 (Ngn3) [1], [2]. Ngn3^+^ cells also give rise to epsilon cells expressing ghrelin, which disappear around 10 days after birth in mice [3]. A hierarchy of transcription factors orchestrates the formation of endocrine cells from Ngn3^+^ progenitors, and mutations in such factors perturb or skew the specification of endocrine cell types. The mechanisms that control the formation of endocrine cells are under intense investigation, in part in the context of efforts to generate transplantable beta cells from embryonic stem cells for the treatment of diabetes.

In parallel to its function in the developing pancreas, Ngn3 controls the formation of enteroendocrine cells in the gastrointestinal tract, which secrete, among others, the hormones secretin, gastrin, GIP, GLP, somatostatin and CCK [4], [5]. While Ngn3 appears to be a master regulator of the generic gut/pancreas endocrine program, it is not clear why different hormones are produced by the pancreatic and the intestinal derivatives of Ngn3^+^ cells. Here we focus on gastrin, a hormone secreted from endocrine G cells located mainly in the gastric antrum [6]–[8]. The gastrin peptide induces acid secretion and gastric motility, and stimulates mucosal proliferation [9]–[11]. Gastric G cells derive from Ngn3^+^ enteroendocrine progenitor cells [4], and their formation requires Nkx2.2 and Arx in addition to Ngn3 [12], [13]. Interestingly, although Ngn3 positive cells are present in the mouse embryonic gut by embryonic day 12.5 [14], the expression of gastrin in the stomach begins only postnataly, so that in fetal life, gastrin is mostly found in the pancreas, in both rodents and humans [15]–[17]. Pancreatic gastrin expression disappears after birth, but can reappear pathologically in the form of gastrin-secreting neuroendocrine tumors (gastrinomas), most of which are malignant [18], [19]. Very little is known about the origins and the molecular determinants of pancreatic gastrinomas and fetal pancreatic gastrin expression. Here we use a combination of expression analysis, genetic lineage tracing and gene knockouts to study gastrin expression in the embryonic pancreas. We demonstrate that G cells represent a distinct, 6th endocrine cell type in the embryonic pancreas, and an alternative fate of Ngn3 endocrine progenitor cells.

## Results

### Expression of gastrin in the embryonic pancreas

**Figure 1:**
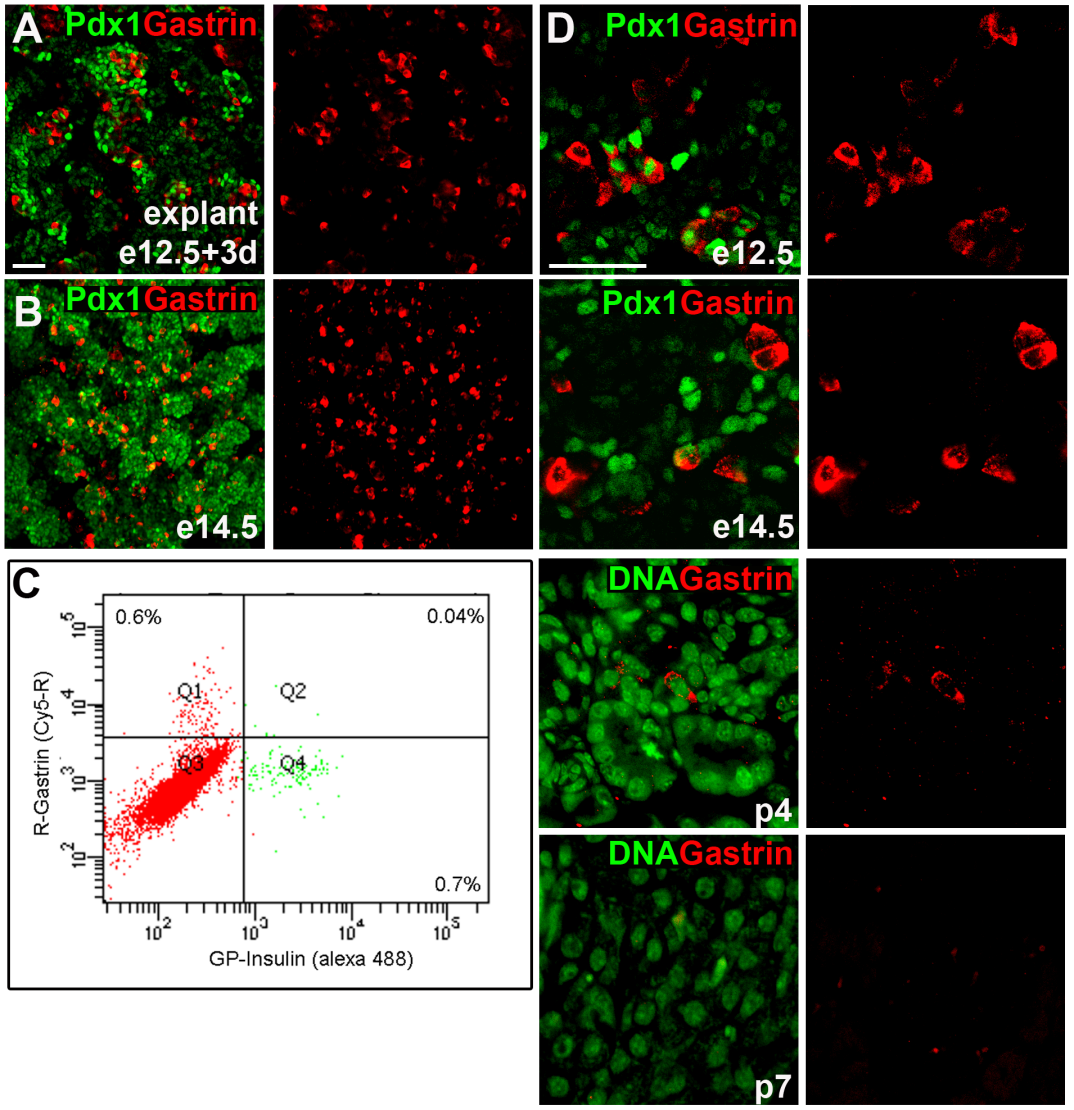
Gastrin is abundantly expressed in the embryonic pancreas, and disappears after birth. A. Expression of gastrin in e12.5 mouse pancreas explanted for 3 days. The image was generated by merging 4 confocal slices (Z-stack). Scale bar = 100 µm. B. In vivo expression of gastrin throughout the pancreas of an e14.5 mouse embryo. The image was generated by merging 11 confocal slices (Z-stack). C. Quantification of gastrin and insulin expression in E14.5 mouse embryo, using flow cytometry after intracellular staining for hormones. X-axis shows relative intensity of flurophore labeling insulin, whereas the y axis shows relative intensity of flurophore labeling gastrin. 0.6% of the cells label gastrin, but not insulin (quadrant Q1), and 0.7% of the cells are labeled with insulin, but not gastrin (quadrant Q4). The 0.04% of cells showing double labeling likely reflects background. D. Expression of gastrin in the mouse pancreas at different developmental ages. All images were taken on a Nikon C1 confocal microscope at a 20× or 60× magnification. Scale bar, 100 µm.

To study gastrin expression in the pancreas at high resolution, we used immunostaining and confocal microscopy. In adult mice, gastrin^+^ cells were observed as expected in the stomach but not in the pancreas (not shown). Consistent with previous reports on gastrin mRNA expression [17], we observed a particularly dramatic abundance of gastrin^+^ cells in explants of e12.5 pancreata cultured for 3 days (Fig. 1A). This observation was repeated in e14.5 embryonic pancreas where numerous gastrin^+^ cells were seen (Fig. 1B). By immunofluorescence, in both samples, gastrin^+^ cells appeared to be nearly as abundant as insulin^+^ cells. FACS analysis of dissociated embryonic pancreata confirmed this observation, demonstrating that 0.6% of the cells expressed gastrin while 0.7% expressed insulin; Fig. 1C and Fig. S1). Gastrin expression declined with embryonic age, essentially disappearing by post-natal day 7 (Fig. 1D).

**Figure 2:**
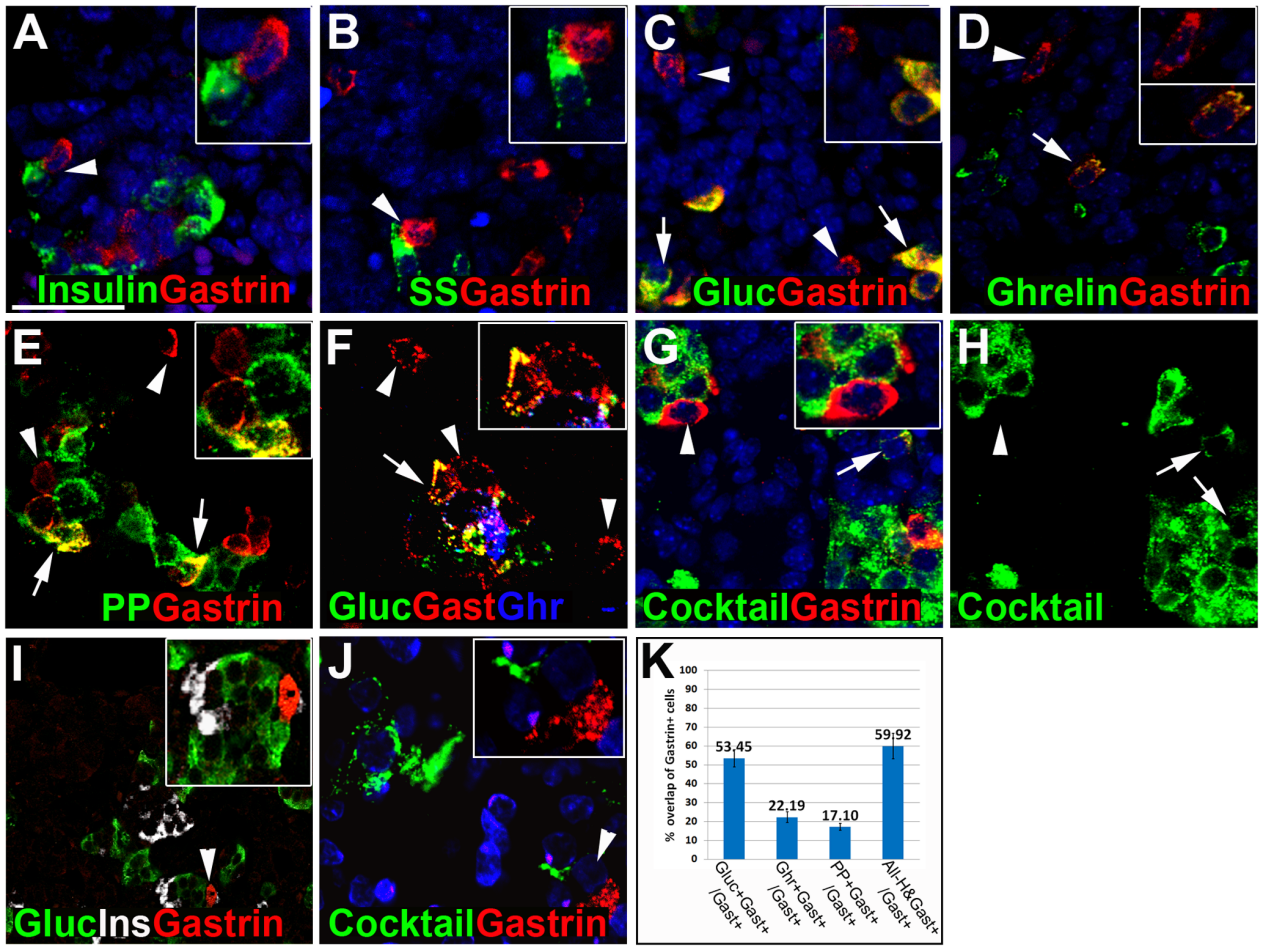
Presence of gastrin-expressing cells that do not stain for other islet hormones. Images in panels A–H are from E15.5 mouse embryos. Arrows indicate cells co-expressing gastrin and other hormones, whereas arrowheads represent cells that express only gastrin. A–E. Co-staining for gastrin and insulin (A) or somatostatin (B) reveals no overlap. Co-staining for gastrin and glucagon (C), ghrelin (D) and pancreatic polypeptide (E) reveals some overlap, but some gastrin positive cells are negative for the other hormones. F. Triple staining for glucagon (green), gastrin (red) and ghrelin (blue) reveals some cells that stain only for gastrin. G–H. Co-staining for gastrin (red) and a cocktail of antibodies against insulin, glucagon, somatostatin, pancreatic polypeptide and ghrelin (G, H, green) reveals some cells that stain only for gastrin. I. Staining for gastrin (DAKO) reveals some gastrin^+^ cells that are negative for insulin and glucagon in e22 Psammomys obesus. J. Co-staining for gastrin (red) and a cocktail of antibodies against insulin, glucagon, somatostatin, pancreatic polypeptide and ghrelin (green) in e22 Psammomys obesus, reveals cells that stain only for gastrin. K. Quantification of cells expressing gastrin with glucagon, ghrelin, pancreatic polypeptide or all pancreatic hormenos as percentage of all cells expressing gastrin at E15.5 (at least 100 cells were counted from different pancreata (n>5)). Scale bar, 100 µm. Values are presented as mean ± SE.

We then characterized the expression of gastrin in the e15.5 mouse embryonic pancreas, and determined the specific cell type expressing gastrin. Co-staining for gastrin and each islet hormone revealed that gastrin^+^ cells did not co-express insulin or somatostatin (Fig. 2A, B). In contrast, gastrin^+^ cells often co-expressed glucagon (53%), ghrelin (22%) or pancreatic polypeptide (17%) (Fig. 2C–E and 2K). Glucagon and ghrelin are co-expressed in endocrine cells during pancreas development. In order to determine whether there are gastrin+ cells that do not co-express these two hormones we co-stained for gastrin, glucagon and ghrelin and found gastrin+ cells that did not express ghrelin or glucagon (Fig. 2F). To further test if there were gastrin^+^ cells that did not express any of the known pancreatic hormones, we co-stained for gastrin and a cocktail of antibodies directed against insulin, glucagon, ghrelin, pancreatic polypeptide and somatostatin. As shown in Fig. 2G–H and Fig. S2, there were many gastrin^+^ cells that did not express any other pancreatic hormone (40%, Fig. 2K). Similar results were obtained when a different gastrin antibody was used to stain the pancreas of the gerbil, *Psammomys obesus*, demonstrating that our findings are not limited to mice. At e22 (equivalent to mouse e15.5), gastrin^+^ cells were readily identified which did not stain for either insulin or glucagon (Fig. 2I). Additionally, we stained e22 *Psammomys obesus* pancreata with the same cocktail used on mouse pancreata and observed gastrin^+^ cells that do not express any other pancreatic hormone (Fig. 2J).

Most existing gastrin antibodies, including those used for the above experiments, cross-react with Cholecystokinin (CCK), a similar hormone that shares with gastrin the amino acid sequence that binds the CCKB receptor and has been reported in the embryonic pancreas. However, CCK expression was only reported in mice from gestational day 16 and beyond, and nearly all CCK^+^ cells co-express glucagon [6], suggesting that our immunostaining identified gastrin^+^ and not CCK^+^ cells. To further confirm this, we used an antibody that does not cross react with CCK (Abnova) and, using this specific antibody, demonstrated gastrin-expressing cells at e15.5 (Fig. S3). As an additional proof, our mRNA analysis (see below) is specific for gastrin, further validating the identification of gastrin. Taken together, these results show that gastrin^+^ cells represent a distinct hormone cell type generated in the embryonic pancreas.

### Gastrin is expressed in cells positive for Nkx2.2, Nkx6.1 and low Pdx1

**Figure 3:**
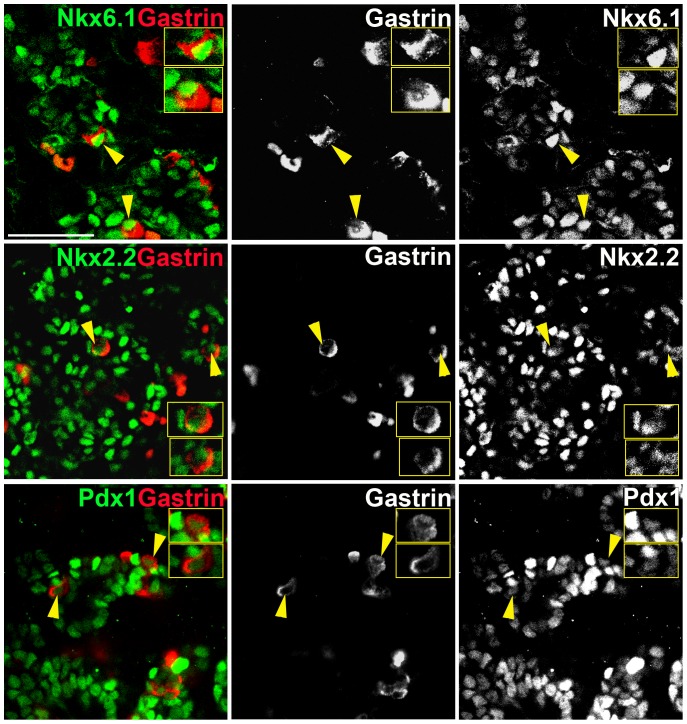
Transcription factors expressed in gastrin^+^ cells. Gastrin^+^ cells in e15.5 pancreas express Nkx6.1, Nkx2.2 and lower levels of Pdx1. Yellow arrows mark the cells in the insets. Scale bar, 100 µm.

To begin and define the transcriptional machinery underlying the genesis of pancreatic gastrin^+^ cells, we co-stained embryonic pancreata for gastrin and key transcription factors. Most gastrin^+^ cells expressed Nkx6.1, Nkx2.2 and stained weakly for Pdx1 (Fig. 3). Gastrin cells never expressed high levels of Pdx1, which is a hallmark of beta-cells.

### Gastrin expressing cells originate from Ngn3^+^ progenitor cells

**Figure 4:**
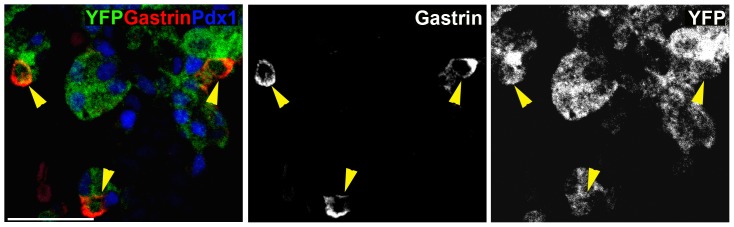
Pancreatic gastrin cells derive from Ngn3+ endocrine progenitors. Co-staining for gastrin (red) and the progeny of Ngn3^+^ cells (anti-GFP, green) in Ngn3-Cre;Rosa26-LSL-YFP e15.5 mice reveals that all gastrin^+^ cells have passed through a Ngn3-expressing stage. Scale bar, 100 µm.

**Figure 5:**
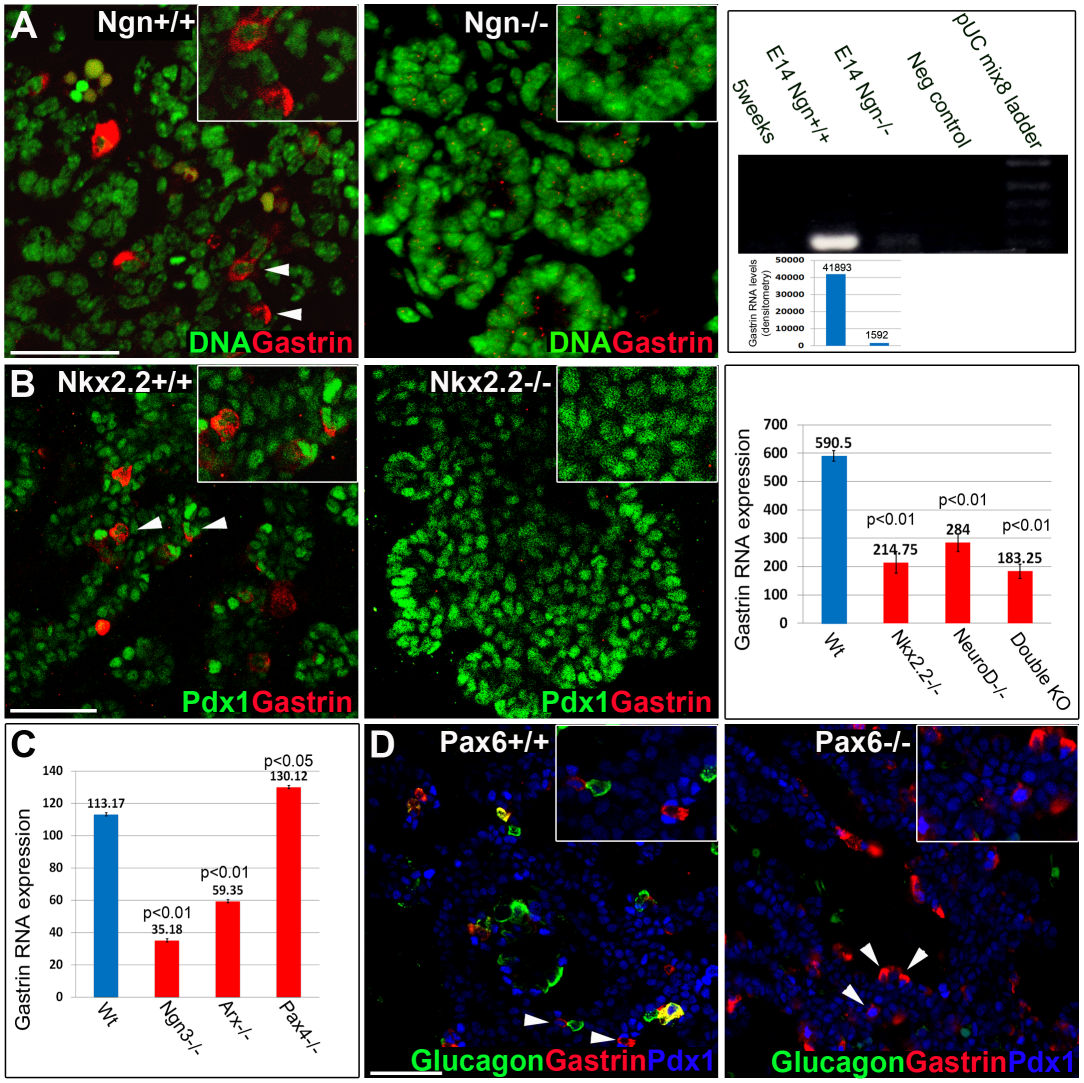
Transcription factors required for gastrin expression in the embryonic pancreas. A. No expression of gastrin in Ngn3^−/−^ pancreata is shown by immunofluorescence staining and significant gastrin reduction by RT-PCR at e14.5. Densitometry measurement of gastrin RNA levels (imageJ) shows that gastrin expression in ngn3^+/+^ pancreata is markedly reduced in ngn3 KO (from 41,893 to 1,592; arbitrary units, the same area was picked for both bands). B. No expression of gastrin in Nkx2.2^−/−^ pancreata is shown by immunofluorescence staining. Microarray analysis shows reduced expression of gastrin RNA in Nkx2.2^−/−^, NeuroD1^−/−^ and double knockout embryos at e14.5. Values are presented as mean ± SE. C. Reduced expression of gastrin in Ngn3^−/−^ and Arx^−/−^ but not in Pax4^−/−^ embryos at e12.5 (microarray analysis). Values are presented as mean ± SE. D. Pax6^−/−^ embryonic pancreata have reduced numbers of gastrin^+^ glucagon^+^ cells, but gastrin^+^ glucagon− cells are still observed. Scale bar, 100 µm for all panels.

Genetic lineage tracing studies have shown that Ngn3^+^ cells give rise to all endocrine cells in the pancreas [1], as well as most enteroendocrine cells in the intestine, including G cells [5]. To determine if gastrin^+^ cells derived from Ngn3-expressing progenitors, we used transgenic mice that express Cre recombinase driven by a BAC containing the Ngn3 promoter, combined with an indelible Cre reporter transgene (*ngn3*-Cre; Rosa26-LSL-YFP). Co-staining of e15.5 pancreata for YFP and gastrin revealed that all gastrin^+^ cells are YFP^+^ (Fig. 4). This result indicates that all gastrin+ cells in the embryonic pancreas are progeny of cells that previously expressed Ngn3. Additionally, examination of *ngn3* ^−/−^ pancreata at e15.5 showed no staining for gastrin, in contrast to an abundant signal in wild type littermates (Fig. 5A). Similar results were obtained in RT-PCR densitometry analysis of RNA extracted from wild type (41,893 arbitrary units) and *ngn3* ^−/−^ (1,592 arbitrary units) pancreata at e14.5 (Fig. 5A). Significant reduction in gastrin RNA levels was also observed in microarray analysis of *ngn3* ^−/−^ pancreata at e12.5 (Fig. 5C). Thus, gastrin^+^ cells represent a 6^th^ fate of Ngn3^+^ pancreatic endocrine progenitor cells.

### Down-stream transcription factors required for pancreatic gastrin expression

To dissect out the additional genetic requirements for gastrin^+^ cell formation in the developing pancreas, we examined embryonic pancreata from mice deficient for key transcription factors known to affect the formation of specific endocrine cell types.

*nkx2.2*-deficient embryos did not show gastrin staining (Fig. 5B), and microarray analysis of *nkx2.2* ^−/−^ pancreata revealed a 70% decrease in the level of gastrin mRNA compared with wild type. We also observed a decrease in the expression of gastrin mRNA in the pancreas of *neuroD1* mutants, as well as in mice doubly deficient for *nkx2.2* and *neuroD1* (Fig. 5B).

Mice deficient for *arx* showed 70% reduction in the levels of gastrin mRNA (Fig. 5C), in contrast to *pax4* ^−/−^ embryos, which had a slight elevation in gastrin levels compared to wild type (Fig. 5C). However *pax4^−/−^* embryos did not reveal any elevation in gastrin by immunostaining (data not shown). Finally, we examined *pax6* ^−/−^ embryos. These mutants had no glucagon+ gastrin+ cells (presumably reflecting the requirement for Pax6 in the formation of alpha cells), but glucagon^−^ gastrin+ cells could be observed in normal numbers, indicating that Pax6 is not necessary for the formation of G cells in the pancreas (Fig. 5D).

These results indicate that gastrin expression in the embryonic pancreas is totally dependent on Ngn3, and to a lesser extent dependent on Nkx2.2, NeuroD1 and Arx, but not on Pax4 or Pax6.

### Gastrin is induced in human ES cells directed to differentiate to pancreatic endocrine cells

**Figure 6:**
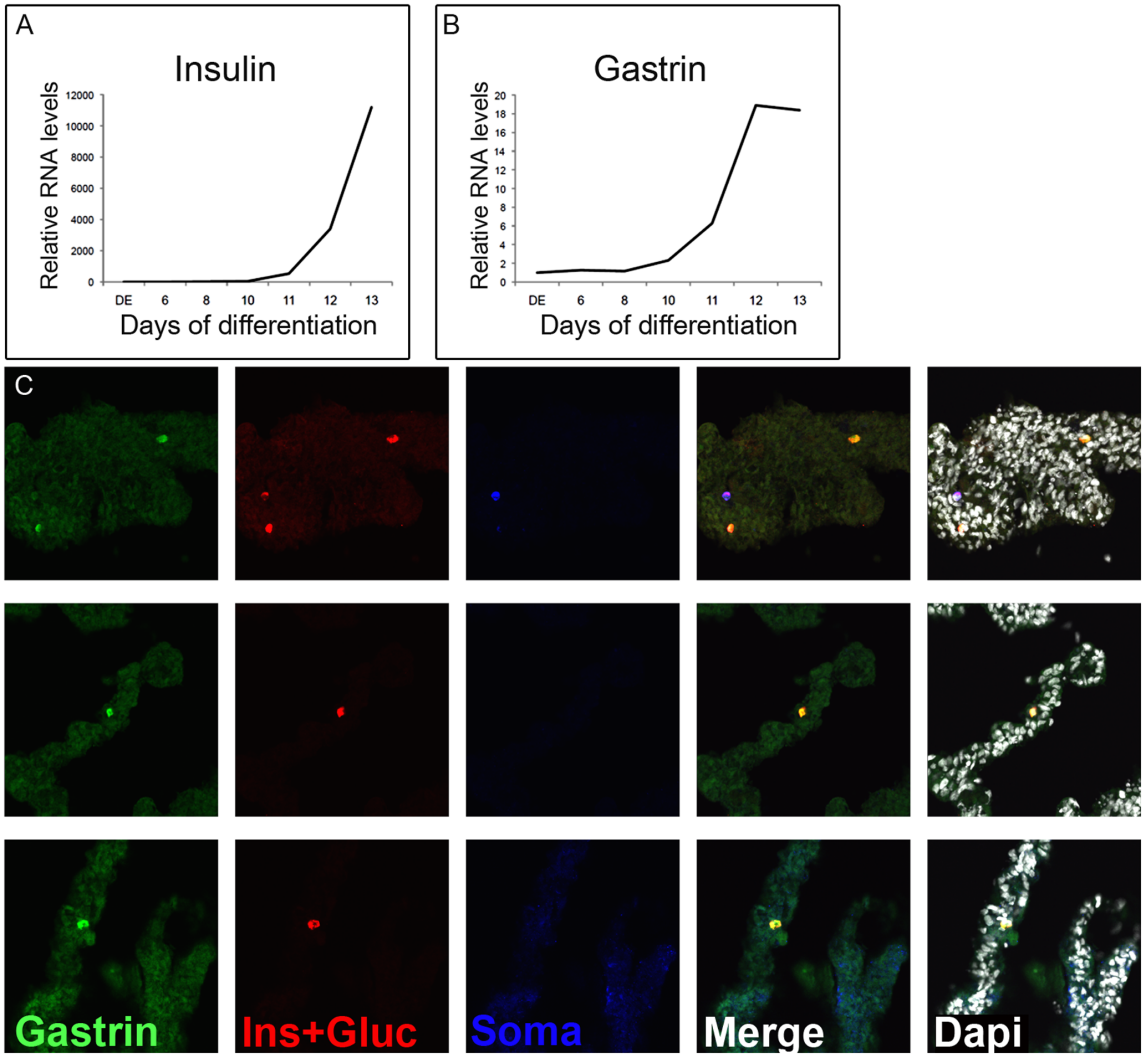
Gastrin expression in human embryonic stem cells. A–B. Quantitative RT-PCR on RNA extracted from human embryonic stem cells, directed to differentiate into definitive endoderm, pancreatic progenitor cells, endocrine progenitor cells, and hormone expressing cells. Note that the kinetics of gastrin induction are similar to those of insulin expression. Values are normalized to beta tubulin and expression differences are relative to definitive endoderm (DE). C. Staining human embryonic stem cells directed to hormone expressing cells for gastrin, insulin, glucagon and somatostatin reveals gastrin positive cells (different samples).

Considerable effort is being invested in the development of protocols for directed differentiation of embryonic stem cells to insulin-producing beta cells that could be transplanted to cure diabetes. Most of these protocols rely on step-wise differentiation aimed to recapitulate embryonic development of beta cells, from definitive endoderm to pancreatic and endocrine progenitor cells, and finally into hormone-expressing cells. We reasoned that such protocols, which give rise to cultures containing a mix of pancreatic hormone expressing cells, could be generating gastrin-expressing cells as well. To test this idea we performed quantitative RT-PCR analysis on human embryonic stem cells differentiated into pancreatic endocrine cells using a modified method that was similar to published protocols [20], [21]. As shown in Fig. 6, gastrin expression was absent when cells were differentiated into definitive endoderm and pancreatic endoderm (day 8). However, there was a rapid induction of gastrin expression as cells were differentiated into endocrine cells. The kinetics of gastrin expression closely mirrored expression of insulin. Staining for gastrin in these cells reveal gastrin positive cells (Fig. 6C) which all co-expressed other hormones, consistent with the notion that endocrine cells generated in vitro are immature and polyhormonal. This finding suggests that, as in rodent, human ESC-derived pancreatic endocrine progenitor cells are competent to differentiate into gastrin^+^ cells. Since beta-cells are the sought-after product of these protocols it is desirable, and perhaps necessary, to reduce the expression of by-products such as gastrin in order to improve efficiency.

## Discussion

### Pancreatic G cells as a distinct cell type

**Figure 7:**
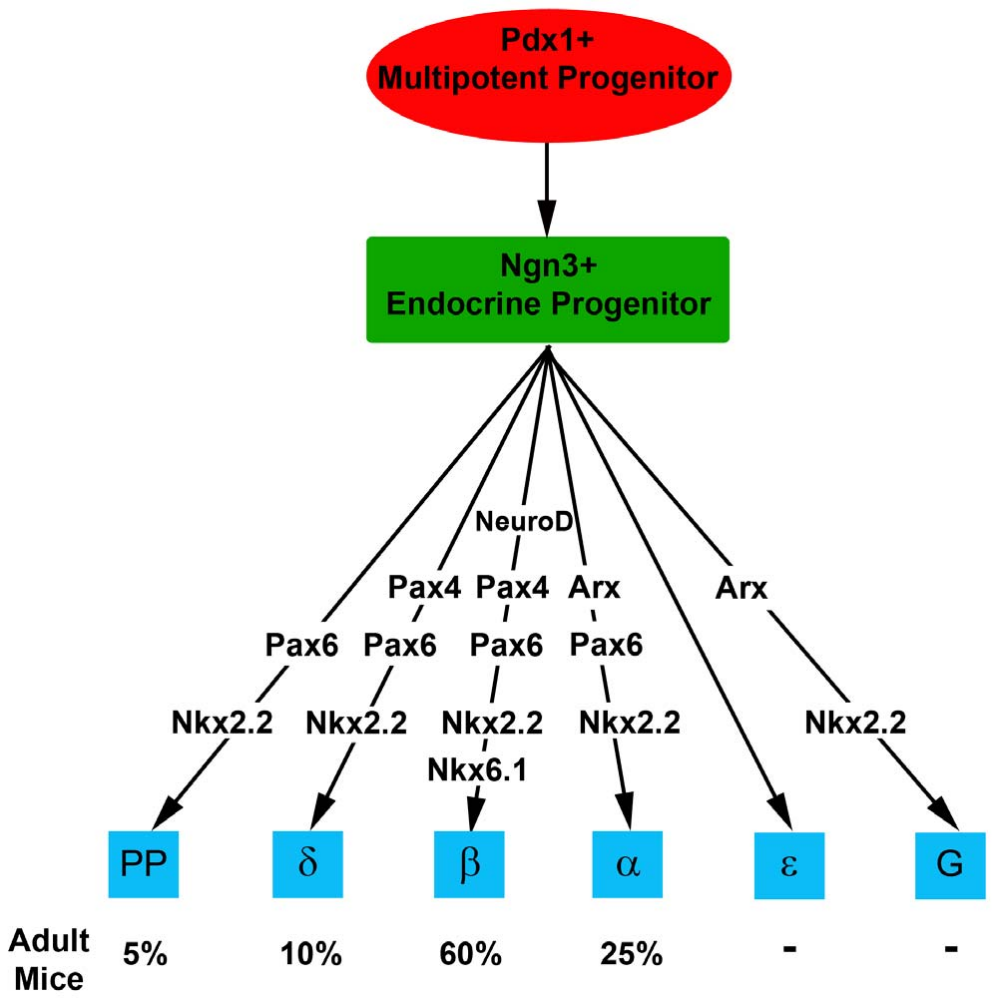
Model placing gastrin cells in the transcriptional hierarchy of endocrine cells in the pancreas.

G cells are well characterized in the adult stomach, where their product, gastrin, serves a clear physiological function - reducing the pH in the lumen of the stomach and increasing stomach motility to accelerate the first steps in digestion of food. Gastric G cells were shown to co-express a number of key transcription factors including Pdx1, Nkx6.1, Nkx6.3 and Arx and to derive from Ngn3^+^ enteroendocrine progenitors. Genetic studies have identified a requirement for Pdx1, Ngn3, Nkx2.2, Pax6 and Arx in the formation of gastric G cells [5], [12],[13],[22]–[24]. It was previously observed that during embryonic development, gastrin mRNA is not expressed in the stomach but rather it is mostly expressed in the developing pancreas, in both rodents and humans [16], [17]. However the significance of this expression pattern received little attention. Here we have performed a detailed analysis of gastrin expression in the embryonic pancreas. Consistent with previous reports, we found that gastrin is abundantly expressed in the embryonic pancreas and disappears soon after birth. Gastrin is co-expressed in some embryonic alpha, epsilon and PP cells, but was never observed in beta or delta cells. Some gastrin^+^ cells appear to express this hormone alone, suggesting that they represent a distinct cell type, the pancreatic G cell. Observations in the gerbil *Psammomys obesus*, using a different gastrin antibody, provide independent support to this conclusion and suggest that gastrin expression is an evolutionary conserved aspect of pancreas development. At the molecular level, we found that pancreatic G cells derive from Ngn3^+^ progenitors, and express Nkx2.2, Nkx6.1 and low levels of Pdx1. We demonstrate using genetic models that gastrin expression in the fetal pancreas depends on Ngn3, Nkx2.2 and Arx (and to a lesser extent on NeuroD1) but not Pax4 or Pax6. Together, these findings identify pancreatic G cells as a distinct endocrine cell type in the pancreas, adding to the 5 other known endocrine cell types formed in this organ (see model, Fig. 7).

The genetic program of gastrin expression in the pancreas is similar to that of alpha cells in that it is dependent on Arx, but also has unique features best illustrated by the formation of G cells in the pancreas of Pax6^−/−^ embryos, which lack alpha cells. Pancreatic G cells also resemble epsilon cells in that both gastrin and ghrelin are frequently co-expressed with glucagon in the embryonic pancreas and the expression of both shifts postnataly from pancreas to stomach. We speculate that these patterns relate to the evolutionary history of the endocrine system. Glucagon and ghrelin may represent the most ancient hormones responding to hunger, which later evolved into the multitude of more “modern” hormones with each developing its own, distinct gut/pancreas location. More work will be required to understand these dynamics. Our finding that protocols currently used for ES cell differentiation result in the production of G-cells provides evidence that these protocols recapitulate embryonic development and enrich for immature, fetal-type endocrine cells. Further steps will likely be needed to complete maturation to adult beta cells.

### Significance of pancreatic gastrin expression

The function of gastrin in the embryonic pancreas is not known. Early studies have suggested that gastrin acts as a mitogen to fetal beta cells [25]–[27], but later studies found little support for this hypothesis. The issue was again raised more recently, with reports that gastrin may induce beta-cell regeneration alone, after 95% pancreatectomy [27], or if co-administered with EGF in the NOD mouse model [28], [29]. However, gastrin-deficient mice have no significant pancreatic phenotype [30] and transgenic overexpression of gastrin in beta cells also resulted in normal pancreatic development [31]. Thus, while pharmacologic administration may have an effect in some circumstances, the physiologic transient pancreatic expression of gastrin may serve no function and, as noted above, may merely reflect the evolutionary history of the endocrine system, similar to pancreatic ghrelin expression and primary transition cells [32].

We show that the expression of gastrin is detected already at e12.5. An interesting possibility is that gastrin+ cells may originate from alpha, epsilon or PP cells. We cannot exclude this possibility but believe it is unlikely. One argument is that it was previously reported that in pax6 knockout the expression of glucagon is abolished. We show that on similar pax6 knockout background the expression of gastrin is not affected.

During adult life, gastrin can be re-expressed in the pancreas in the form of functional islet cell tumors, ∼40% of which are gastrinomas [33]–[35]. The cell of origin of pancreatic gastrinomas remains unknown. They could derive from rare gastrin^+^ cells that survived in the pancreas from fetal life; from reprogramming of adult islet cells that undergo malignant transformation; or from aberrant postnatal activation of the embryonic program of endocrine differentiation. Genetic lineage tracing studies will be needed to address this problem; such studies may also reveal whether embryonic G cells are eliminated, or whether they downregulate gastrin expression and resolve to another cell type. However, such experiments are beyond the scope of this study. Finally, pancreatic islet tumors rarely express additional hormones such as Vasoactive Intestinal Polypeptide (VIP) and parathyroid hormone-related peptide (PTHRP). It will be interesting to test whether, like gastrin, these hormone are transiently expressed during normal pancreas development.

### Determinants of islet cell composition

Our data on embryonic gastrin expression, together with the similar dynamics of ghrelin expression, highlight a striking difference between the composition and relative abundance of endocrine cells in the embryonic and adult pancreas. While Ngn3^+^ cells generate 6 different cell types in the fetal endocrine pancreas, postnatal processes remodel this starting population to obtain a different cell composition in the adult islet (Fig. 7). It will be interesting to study how differential rates of proliferation and survival affect this process and the molecular mechanisms that impact postnatal hormone gene expression.

## Materials and Methods

### Ethics Statement

The joint ethics committee (IACUC) of the Hebrew University and Hadassah Medical Center approved the study protocol for animal welfare. The Hebrew University is an AAALAC International accredited institute. Experiments with *P. obesus* were performed in accordance to the regulations specified under the Protection of Animals Act by the authority in Denmark, European Union and Novo Nordisk A/S.

### Mice

We used either ICR mice from Harlan, Israel or knockout strains as previously described: Homozygous *Ngn3*-CreER (serving as Ngn3 nulls) [36], BAC transgenic *Ngn3*-Cre [37], *ROSA26*-LSL-YFP [38], *Nkx2.2^−/−^* [32], *NeuroD^−/−^* [39], *Pax6^−/−^* [40], *Arx^−/−^* [41], *Pax4^−/−^* [42]. Experiments with *P. obesus* were performed in accordance to the regulations specified under the Protection of Animals Act by the authority in Denmark, European Union and Novo Nordisk A/S.

### Pancreatic explants

Pancreatic buds from embryonic day (e) 12.5 mouse embryos were cultured in DMEM supplemented with 10% fetal bovine serum, containing 100 U/ml penicillin and 100 µg/ml streptomycin, as described previously [43]. Explants were cultured on microporous membranes (Whatman, nucleopore Track-Etch, diameter 13 mm, pore size 8.0 micron) and no medium was added on top of the filter, so that the tissue grew at the air/medium interface. Medium was changed every other day.

### Immunostaining, antibodies and microscopy

All images are representative of at least 6 different pancreata.

Whole mount pancreas immunostaining was performed essentially as previously described [44]. For all other immunostaining, pancreata (e12.5-p7) were surgically removed and fixed in 4% buffered zinc-formalin for 2 hours at 4°C, dehydrated in an ethanol series, cleared in histoclear and embedded in Paraplast (Kendall). When required, antigen retrieval was performed using a citrate buffer and a pressure cooker. Primary antibodies used in this study included: Rabbit anti-gastrin (1∶200, Cell Marquee), rabbit anti-gastrin (1∶200, Dako), guinea pig anti-gastrin (1∶50, Progen), mouse anti-gastrin (1∶50, Abnova), guinea pig anti-insulin (1∶200, Abcam), goat anti-pancreatic polypeptide (1∶50, Abcam), mouse anti-glucagon (1∶800, Beta Cell Biology Consortium), mouse anti-somatostatin (1∶400, Beta Cell Biology Consortium), goat anti-ghrelin (1∶100, Santa Cruz), goat anti-GFP (1∶500, Abcam), goat anti-Pdx1 (1∶2,500, a generous gift of Chris Wright), mouse anti-Nkx2.2 (1∶50, Developmental Studies Hybridoma Bank) and mouse anti Nkx6.1 (1∶400, Beta Cell Biology Consortium). For DNA counterstain we used Sytox (1∶500, Invitrogen). Rabbit anti-Gastrin from Cell Marque was used in the images shown unless stated otherwise. Guinea-pig and mouse anti gastrin were used to confirm the rabbit antibody findings. The gastrin antibodies produced by Progen, Cell-Marque and DAKO cross-react with CCK (100, 50, 20% cross-reactivity respectively) whereas the Abnova antibody does not. Secondary antibodies were from Jackson immune-research, used at 1∶200. Immunofluorescence images were captured on a Nikon C1 confocal microscope or Olympus FV1000.

### Flow cytometry

For FACS analysis, e14.5 pancreata or livers from wild type embryos were dissociated to single cells with trypsin and stained with guinea pig anti-insulin (1∶800, Abcam) followed by Alexa488 anti-guinea pig (1∶500, Jackson Immunoresearch), and with Rabbit anti-gastrin (1∶500, Cell Marquee) followed by Cy5 anti-rabbit (1∶800, Jackson Immunoresearch).

### RT-PCR

Total RNA (n = 5) was prepared using Qiagen RNeasy microkit according to the manufacturer's protocol. Total RNA (100 ng) was used for first-strand cDNA synthesis using random primers (Roche) and reverse transcriptase (ImProm-II,Promega). We checked the existence or absence of Gastrin mRNA in wild type embryos, wild type adult pancreas and Ngn3-deficient pancreata. PCR was performed using GoTaq Green Master Mix (Promega). Primers were designed to bridge introns to avoid amplifying genomic DNA contamination if present: mGastrin: F-5′- cccagctctgtggacaagatgcctc-3′ and R-5′- gcatcctgtagctgggagcgg -3′; hB-tubulin F-5′- gatacctcaccgtggctgct -3′ and R-5′- agaggaaaggggcagttgagt – 3′; hInsulin F-5′- gaaccaacacctgtgcggctca - 3′ and R-5′- tgcctgcgggctgcgtctagt - 3′; hGastrin F-5′- cagagccagtgcaaagatca - 3′ and R-5′- agagacctgagaggcaccag - 3′. Note that the gastrin primers do not cross-react with the CCK gene.

### Micro-Array analysis

#### Nkx2.2 and NeuroD

Pancreas tissue was collected from either Nkx2.2^−/−^, NeuroD^−/−^, Nkx2.2^−/−^ NeuroD^−/−^ or WT staged embryos at e14.5 (n = 4). Total pancreata were collected, stored individually in RNase Later (Ambion), and genotyped. Four groups of wild-type and mutant tissues were individually pooled. Samples of total RNA were processed for microarray analysis by using the Mu19k A gene chip according to manufacturer's instructions (Affymetrix). Chip performance, background levels, and presence/absence calls were assessed by using MICROARRAY SUITE software (Affymetrix). The processing of data was performed in GenePattern (Broad Institute).

#### Ngn3, Arx and Pax4

Pancreas tissue was collected from Ngn3−/−, Arx−/−, Pax4−/− or WT staged embryos at e12.5 (n = 3). Total pancreata were collected and samples of total RNA were processed for microarray analysis using Affymetrix protocol for affymetrix 430 arrays. The processing of data was performed in Genesifter.

### Pancreas differentiation from human pluripotent stem cells

Human pluripotent stem cells were differentiated into pancreatic tissue using methods adapted from previous reports [20], [21]. The human embryonic stem cell line WA09 (WiCell) was maintained in feeder-free culture conditions on Matrigel (BD Biosciences) in mTesR1 medium (Stem Cell Technologies). For differentiation, cells were treated with Dispase (Invitrogen) and plated into a 24-well plate. For definitive endoderm induction, 80–90% confluent cells were treated with Activin A (100 ng/ml; R&D Systems) for three days in RPMI 1640 medium (Invitrogen) containing 0%, 0.2%, and 2.0% dFBS (Thermo Scientific) on days 1, 2, and 3. The definitive endoderm was further cultured for two days in the presence of FGF10 (50 ng/ml; R&D Systems) and noggin (50 ng/ml; R&D Systems) in RPMI 1640+2.0% dFBS. For induction of pancreatic endoderm, cells were then treated for four days with all-trans retinoic acid (2 µM; Stemgent), KAAD (0.25 µM; Stemgent), FGF10 (50 ng/ml), and noggin (50 ng/ml) in DMEM medium (Invitrogen) with B27 supplement (2%; Invitrogen). This was followed by five days of culture in EGF (50 ng/ml; R&D Systems) in DMEM/B27, and then six days in Exendin-4 (50 ng/ml; Tocris Bioscience) and DAPT (10 µM; Stemgent) in DMEM/B27.

### Statistics

Values are presented as mean ± SE. P values were determined using the 2-tailed Student's t test with unequal variance. P values of less than 0.05 were considered significant. ***, p<0.001, **, p<0.01, * p<0.05.

## Supporting Information

Figure S1 **Controls for FACS analysis quantifying gastrin^+^ cells in the embryonic pancreas.** Left panels: e14.5 pancreata from wild type mice were dissociated to single cells, stained with fluorescent antibodies against either insulin or gastrin and analyzed by FACS. The panels show that in these preps, 0.7% of pancreatic cells are insulin+ and 0.6% are gastrin+ cells. Right panels: e14.5 livers from wild type mice were dissociated to single cells, stained with fluorescent antibodies against insulin and gastrin and analyzed by FACS. The panels show that liver cells are negative both for insulin and gastrin antibodies suggesting for specificity of our antibodies.
(TIF)

Figure S2 **More evidence for endocrine cells in the embryonic pancreas that express only gastrin.** Sections of e14.5 pancreata co-stained for gastrin (red) and a cocktail of antibodies against insulin, glucagon, somatostatin, pancreatic polypeptide and ghrelin (green). Some cells that stain only for gastrin, while some co-express gastrin and other hormones (appearing in yellow).
(TIF)

Figure S3 **Gastrin expressing cells do not stain for CCK.** Sections of several e15.5 pancreata stained with an antibody specific for gastrin that does not cross react with CCK (Abnova).
(TIF)

## References

[1] Gradwohl G, Dierich A, LeMeur M, Guillemot F (2000) neurogenin3 is required for the development of the four endocrine cell lineages of the pancreas. Proc Natl Acad Sci U S A 97: 1607–1611. 10677506 10.1073/pnas.97.4.1607 PMC26482

[2] Gu G, Dubauskaite J, Melton DA (2002) Direct evidence for the pancreatic lineage: NGN3+ cells are islet progenitors and are distinct from duct progenitors. Development 129: 2447–2457. 11973276 10.1242/dev.129.10.2447

[3] Heller RS, Jenny M, Collombat P, Mansouri A, Tomasetto C, et al (2005) Genetic determinants of pancreatic epsilon-cell development. Dev Biol 286: 217–224. 16122727 10.1016/j.ydbio.2005.06.041

[4] Jenny M, Uhl C, Roche C, Duluc I, Guillermin V, et al (2002) Neurogenin3 is differentially required for endocrine cell fate specification in the intestinal and gastric epithelium. Embo J 21: 6338–6347. 12456641 10.1093/emboj/cdf649 PMC136953

[5] Lee CS, Perreault N, Brestelli JE, Kaestner KH (2002) Neurogenin 3 is essential for the proper specification of gastric enteroendocrine cells and the maintenance of gastric epithelial cell identity. Genes Dev 16: 1488–1497. 12080087 10.1101/gad.985002 PMC186338

[6] Dockray GJ, Varro A, Dimaline R, Wang T (2001) The gastrins: their production and biological activities. Annu Rev Physiol 63: 119–139. 11181951 10.1146/annurev.physiol.63.1.119

[7] Sawada M, Dickinson CJ (1997) The G cell. Annu Rev Physiol 59: 273–298. 9074765 10.1146/annurev.physiol.59.1.273

[8] Soll AH, Yamada T, Park J, Thomas LP (1984) Release of somatostatinlike immunoreactivity from canine fundic mucosal cells in primary culture. Am J Physiol 247: G558–566. 6149697 10.1152/ajpgi.1984.247.5.G558

[9] Baldwin GS (1995) The role of gastrin and cholecystokinin in normal and neoplastic gastrointestinal growth. J Gastroenterol Hepatol 10: 215–232. 7787172 10.1111/j.1440-1746.1995.tb01083.x

[10] Logan CJ, Connell AM (1966) The effect of a synthetic gastrin-like pentapeptide (I.C.I. 50,123) on intestinal motility in man. Lancet 1: 996–999. 4161114 10.1016/s0140-6736(66)90111-5

[11] Samuelson LC, Hinkle KL (2003) Insights into the regulation of gastric acid secretion through analysis of genetically engineered mice. Annu Rev Physiol 65: 383–400. 12517996 10.1146/annurev.physiol.65.092101.142213

[12] Desai S, Loomis Z, Pugh-Bernard A, Schrunk J, Doyle MJ, et al (2008) Nkx2.2 regulates cell fate choice in the enteroendocrine cell lineages of the intestine. Dev Biol 313: 58–66. 18022152 10.1016/j.ydbio.2007.09.047 PMC2238176

[13] Du A, McCracken KW, Walp ER, Terry NA, Klein TJ, et al (2012) Arx is required for normal enteroendocrine cell development in mice and humans. Dev Biol 365: 175–188. 22387004 10.1016/j.ydbio.2012.02.024 PMC3322318

[14] Kokubu H, Ohtsuka T, Kageyama R (2008) Mash1 is required for neuroendocrine cell development in the glandular stomach. Genes Cells 13: 41–51. 18173746 10.1111/j.1365-2443.2007.01146.x

[15] Takaishi S, Shibata W, Tomita H, Jin G, Yang X, et al (2011) In vivo analysis of mouse gastrin gene regulation in enhanced GFP-BAC transgenic mice. Am J Physiol Gastrointest Liver Physiol 300: G334–344. 21051525 10.1152/ajpgi.00134.2010 PMC3043646

[16] Bardram L, Hilsted L, Rehfeld JF (1990) Progastrin expression in mammalian pancreas. Proc Natl Acad Sci U S A 87: 298–302. 2296587 10.1073/pnas.87.1.298 PMC53250

[17] Gittes GK, Rutter WJ, Debas HT (1993) Initiation of gastrin expression during the development of the mouse pancreas. Am J Surg 165: 23–25 discussion 25-26. 8418699 10.1016/s0002-9610(05)80399-x

[18] Bertolino P, Tong WM, Galendo D, Wang ZQ, Zhang CX (2003) Heterozygous Men1 mutant mice develop a range of endocrine tumors mimicking multiple endocrine neoplasia type 1. Mol Endocrinol 17: 1880–1892. 12819299 10.1210/me.2003-0154

[19] Gregory RA, Tracy HJ, French JM, Sircus W (1960) Extraction of a gastrin-like substance from a pancreatic tumour in a case of Zollinger-Ellison syndrome. Lancet 1: 1045–1048. 13851712 10.1016/s0140-6736(60)90932-6

[20] D'Amour KA, Bang AG, Eliazer S, Kelly OG, Agulnick AD, et al (2006) Production of pancreatic hormone-expressing endocrine cells from human embryonic stem cells. Nat Biotechnol 24: 1392–1401. 17053790 10.1038/nbt1259

[21] Mfopou JK, Chen B, Sui L, Sermon K, Bouwens L (2010) Recent advances and prospects in the differentiation of pancreatic cells from human embryonic stem cells. Diabetes 59: 2094–2101. 20805383 10.2337/db10-0439 PMC2927928

[22] Choi MY, Romer AI, Wang Y, Wu MP, Ito S, et al (2008) Requirement of the tissue-restricted homeodomain transcription factor Nkx6.3 in differentiation of gastrin-producing G cells in the stomach antrum. Mol Cell Biol 28: 3208–3218. 18347062 10.1128/MCB.01737-07 PMC2423174

[23] Larsson LI, Madsen OD, Serup P, Jonsson J, Edlund H (1996) Pancreatic-duodenal homeobox 1 -role in gastric endocrine patterning. Mech Dev 60: 175–184. 9025070 10.1016/s0925-4773(96)00609-0

[24] Larsson LI, St-Onge L, Hougaard DM, Sosa-Pineda B, Gruss P (1998) Pax 4 and 6 regulate gastrointestinal endocrine cell development. Mech Dev 79: 153–159. 10349628 10.1016/s0925-4773(98)00182-8

[25] Bouwens L, Rooman I (2005) Regulation of pancreatic beta-cell mass. Physiol Rev 85: 1255–1270. 16183912 10.1152/physrev.00025.2004

[26] Rooman I, Lardon J, Bouwens L (2002) Gastrin stimulates beta-cell neogenesis and increases islet mass from transdifferentiated but not from normal exocrine pancreas tissue. Diabetes 51: 686–690. 11872667 10.2337/diabetes.51.3.686

[27] Tellez N, Joanny G, Escoriza J, Vilaseca M, Montanya E (2011) Gastrin treatment stimulates beta-cell regeneration and improves glucose tolerance in 95% pancreatectomized rats. Endocrinology 152: 2580–2588. 21558313 10.1210/en.2011-0066

[28] Suarez-Pinzon WL, Yan Y, Power R, Brand SJ, Rabinovitch A (2005) Combination therapy with epidermal growth factor and gastrin increases beta-cell mass and reverses hyperglycemia in diabetic NOD mice. Diabetes 54: 2596–2601. 16123347 10.2337/diabetes.54.9.2596

[29] Wang M, Racine JJ, Song X, Li X, Nair I, et al (2012) Mixed chimerism and growth factors augment beta cell regeneration and reverse late-stage type 1 diabetes. Sci Transl Med 4: 133ra159. 10.1126/scitranslmed.3003835 22572882

[30] Wang TC, Dockray GJ (1999) Lessons from genetically engineered animal models. I. Physiological studies with gastrin in transgenic mice. Am J Physiol 277: G6–11. 10409145 10.1152/ajpgi.1999.277.1.G6

[31] Wang TC, Bonner-Weir S, Oates PS, Chulak M, Simon B, et al (1993) Pancreatic gastrin stimulates islet differentiation of transforming growth factor alpha-induced ductular precursor cells. J Clin Invest 92: 1349–1356. 8376589 10.1172/JCI116708 PMC288276

[32] Sussel L, Kalamaras J, Hartigan-O'Connor DJ, Meneses JJ, Pedersen RA, et al (1998) Mice lacking the homeodomain transcription factor Nkx2.2 have diabetes due to arrested differentiation of pancreatic beta cells. Development 125: 2213–2221. 9584121 10.1242/dev.125.12.2213

[33] Carty SE, Helm AK, Amico JA, Clarke MR, Foley TP, et al (1998) The variable penetrance and spectrum of manifestations of multiple endocrine neoplasia type 1. Surgery 124: 1106–1113 discussion 1113-1104. 9854591 10.1067/msy.1998.93107

[34] Hao W, Skarulis MC, Simonds WF, Weinstein LS, Agarwal SK, et al (2004) Multiple endocrine neoplasia type 1 variant with frequent prolactinoma and rare gastrinoma. J Clin Endocrinol Metab 89: 3776–3784. 15292304 10.1210/jc.2003-031511

[35] Samaan NA, Ouais S, Ordonez NG, Choksi UA, Sellin RV, et al (1989) Multiple endocrine syndrome type I. Clinical, laboratory findings, and management in five families. Cancer 64: 741–752. 2568165 10.1002/1097-0142(19890801)64:3<741::aid-cncr2820640329>3.0.co;2-f

[36] Wang S, Yan J, Anderson DA, Xu Y, Kanal MC, et al (2010) Neurog3 gene dosage regulates allocation of endocrine and exocrine cell fates in the developing mouse pancreas. Dev Biol 339: 26–37. 20025861 10.1016/j.ydbio.2009.12.009 PMC2824035

[37] Schonhoff SE, Giel-Moloney M, Leiter AB (2004) Neurogenin 3-expressing progenitor cells in the gastrointestinal tract differentiate into both endocrine and non-endocrine cell types. Dev Biol 270: 443–454. 15183725 10.1016/j.ydbio.2004.03.013

[38] Srinivas S, Watanabe T, Lin CS, William CM, Tanabe Y, et al (2001) Cre reporter strains produced by targeted insertion of EYFP and ECFP into the ROSA26 locus. BMC Dev Biol 1: 4. 11299042 10.1186/1471-213X-1-4 PMC31338

[39] Miyata T, Maeda T, Lee JE (1999) NeuroD is required for differentiation of the granule cells in the cerebellum and hippocampus. Genes Dev 13: 1647–1652. 10398678 10.1101/gad.13.13.1647 PMC316850

[40] St-Onge L, Sosa-Pineda B, Chowdhury K, Mansouri A, Gruss P (1997) Pax6 is required for differentiation of glucagon-producing alpha-cells in mouse pancreas. Nature 387: 406–409. 9163426 10.1038/387406a0

[41] Collombat P, Mansouri A, Hecksher-Sorensen J, Serup P, Krull J, et al (2003) Opposing actions of Arx and Pax4 in endocrine pancreas development. Genes Dev 17: 2591–2603. 14561778 10.1101/gad.269003 PMC218152

[42] Sosa-Pineda B, Chowdhury K, Torres M, Oliver G, Gruss P (1997) The Pax4 gene is essential for differentiation of insulin-producing beta cells in the mammalian pancreas. Nature 386: 399–402. 9121556 10.1038/386399a0

[43] Magenheim J, Ilovich O, Lazarus A, Klochendler A, Ziv O, et al (2011) Blood vessels restrain pancreas branching, differentiation and growth. Development 138: 4743–4752. 21965615 10.1242/dev.066548 PMC4496872

[44] Ahnfelt-Ronne J, Hald J, Bodker A, Yassin H, Serup P, et al (2007) Preservation of proliferating pancreatic progenitor cells by Delta-Notch signaling in the embryonic chicken pancreas. BMC Dev Biol 7: 63. 17555568 10.1186/1471-213X-7-63 PMC1906762

