# Supplementary figures and images for: Gastrin: A Distinct Fate of Neurogenin3 Positive Progenitor Cells in the Embryonic Pancreas
Source: PLoS One. 2013 Aug 5;8(8):e70397. doi: 10.1371/journal.pone.0070397 (PMC3734289; doi:10.1371/journal.pone.0070397)

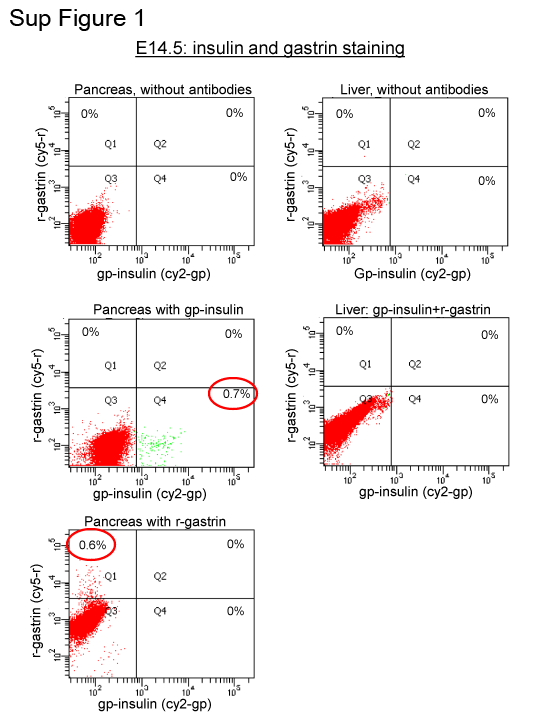

Supplement: Figure S1 — Controls for FACS analysis quantifying gastrin+ cells in the embryonic pancreas. Left panels: e14.5 pancreata from wild type mice were dissociated to single cells, stained with fluorescent antibodies against either insulin or gastrin and analyzed by FACS. The panels show that in these preps, 0.7% of pancreatic cells are insulin+ and 0.6% are gastrin+ cells. Right panels: e14.5 livers from wild type mice were dissociated to single cells, stained with fluorescent antibodies against insulin and gastrin and analyzed by FACS. The panels show that liver cells are negative both for insulin and gastrin antibodies suggesting for specificity of our antibodies. (TIF) [file pone.0070397.s001.tif]

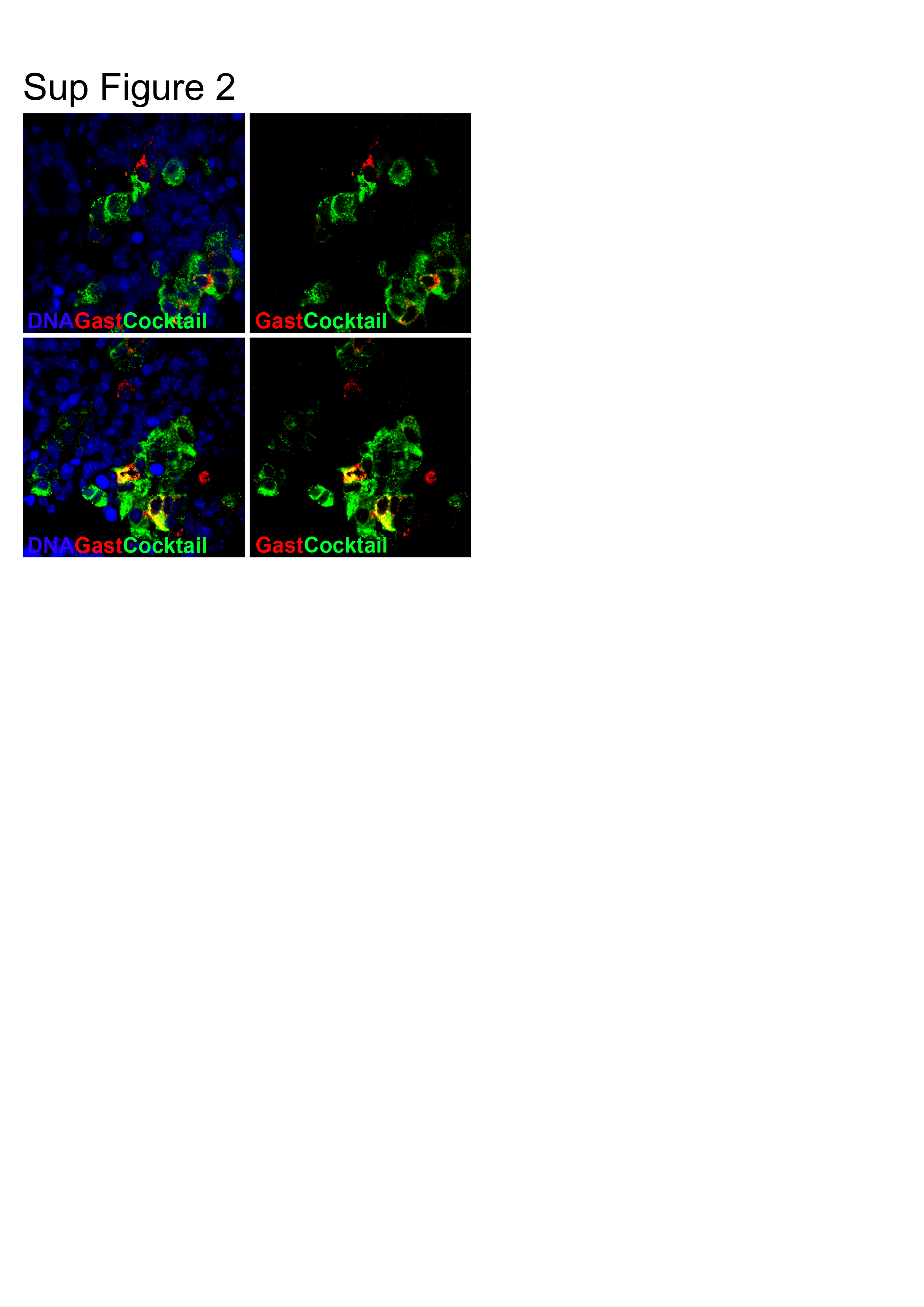

Supplement: Figure S2 — More evidence for endocrine cells in the embryonic pancreas that express only gastrin. Sections of e14.5 pancreata co-stained for gastrin (red) and a cocktail of antibodies against insulin, glucagon, somatostatin, pancreatic polypeptide and ghrelin (green). Some cells that stain only for gastrin, while some co-express gastrin and other hormones (appearing in yellow). (TIF) [file pone.0070397.s002.tif]

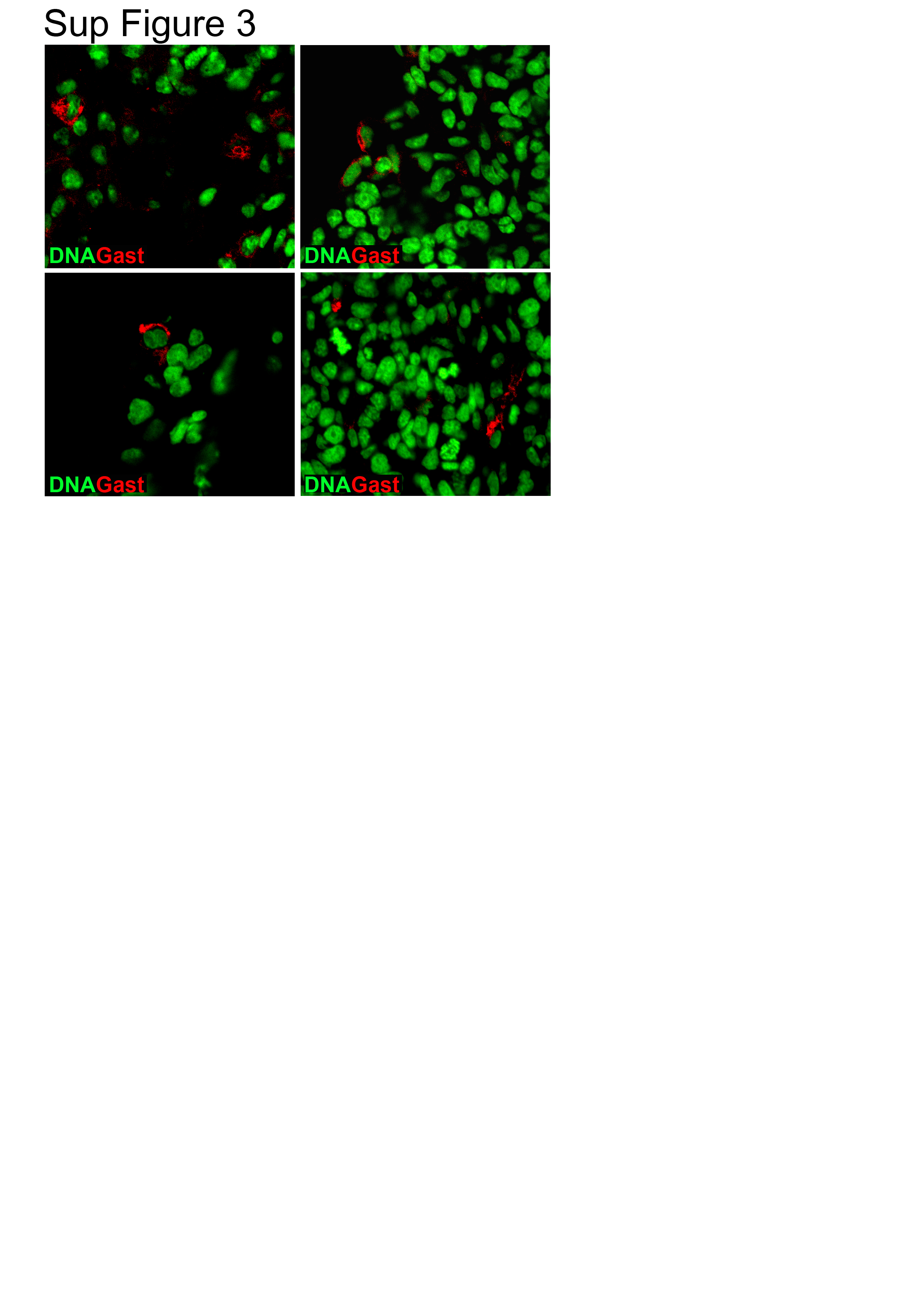

Supplement: Figure S3 — Gastrin expressing cells do not stain for CCK. Sections of several e15.5 pancreata stained with an antibody specific for gastrin that does not cross react with CCK (Abnova). (TIF) [file pone.0070397.s003.tif]
